# Determination of synbiotic mango fruit yogurt and its bioactive peptides for biofunctional properties

**DOI:** 10.3389/fchem.2024.1470704

**Published:** 2025-01-20

**Authors:** Jagrani Minj, Shilpa Vij

**Affiliations:** Dairy Microbiology Division, ICAR-National Dairy Research Institute, Karnal, Haryana, India

**Keywords:** yogurt, synbiotic, antioxidant activity, bioactive peptides, mango fruit yogurt, biofunctional yogurt

## Abstract

Yogurt is one of the most popular fermented milk products consumed worldwide. Fortification of yogurt with different food components, including fruit pulp, is a common practice to make it more palatable and healthier. In India, mango fruit is easily available. It is rich in nutrients and bioactive components. However, in-depth studies on mango fruit yogurt are scarce. Therefore, in this study, we prepared synbiotic mango fruit yogurt using response surface methodology (RSM) with three different independent factors (sugar 4%–6%; prebiotic inulin 1%–3%, and mango pulp 5%–15%) to determine the response antioxidant activity. The optimal conditions were as follows: sugar 6%, mango fruit pulp 6.562%, and prebiotic inulin 1%. There were no significant differences between the results of the experimental and predicted values of antioxidant activity by this model. The optimized product was analyzed for physicochemical, biofunctional, and technofunctional properties, including total polyphenol content, total flavonoid content, proteolytic activity, antioxidant activity, and ACE-inhibitory activity. The bioactive peptides derived from synbiotic mango fruit yogurt were also extracted (3 kDa, 5 kDa and 10 kDa) and determined for their biofunctional attributes. The antioxidant activity was recorded as 1,047.95 ± 2.20 mmol/L, 1,208.07 ± 2.92 mmol/L, and 1,293.09 ± 1.10 mmol/L Trolox equivalent antioxidant capacity, while ACE-inhibitory activity was 45.68% ± 1.23%, 64.20% ± 1.24% and 82.72% ± 1.24% inhibition in 3 kDa, 5 kDa, and 10 kDa, respectively. The 10 kDa bioactive peptide exhibited superior results than the 3 kDa and 5 kDa peptides. The synbiotic mango fruit yogurt and its bioactive peptides showed significant biofunctional activities.

## Graphical Abstract

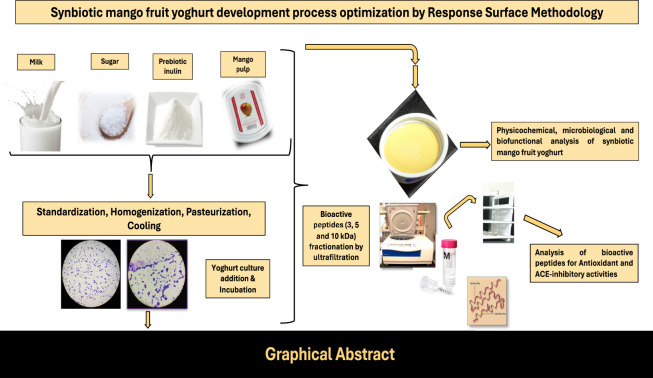

## 1 Introduction

Fermented milk product yogurt is a healthy and nutrient-rich dairy product made by yogurt culture *Lactobacillus bulgaricus* subsp. *delbrueckii* and *Streptococcus thermophilus*. Yogurt can be prepared as plain or fortified types, based on the consumer’s demand. Recently, consumers have become aware of healthy dairy products, including nutritional and biofunctional rich yogurt. The preparation of nutritional and biofunctional rich yogurt requires either incorporation of well-proven probiotic bacteria, prebiotic inulin, or other compatible food components such as fruit pulp, purees, or fruits. The FAO/WHO defines probiotics as “live microorganisms, which, when administered in adequate amounts, confer a health benefit on the host” (Food and Agriculture Organization of the United Nations and World Health Organization, 2006). Probiotic bacteria have many health benefits such as improvement in lactose digestion (EFSA Panel on Dietetic ProductsNutrition and Allergies, 2010), support digestive health system (Sanders et al., 2018), reduction of risk of high blood pressure (Beltrán-Barrientos et al., 2018), improvement on immune system via maintaining gut barrier function (George Kerry et al., 2018) and colonization (O’Flaherty et al., 2010), immunomodulation (Sanders et al., 2013; Bodke and Jogdand, 2022), improvements in antioxidant activity and fasting blood glucose (Ejtahed et al., 2012), improvement in insulin sensitivity (Tonucci et al., 2017), reduction of serum cholesterol (Munir et al., 2022), and many more. The application of probiotic yogurt bacteria along with other probiotic lactic acid bacteria also increases the functionality of the developed product (Minj et al., 2021). Therefore, yogurt prepared with probiotic bacteria is beneficial for overall human health.

The addition of probiotic bacteria along with prebiotic components such as inulin further improves the biofunctional properties of developed yogurt. A prebiotic is defined as “a substrate that is selectively utilized by host microorganisms conferring a health benefit” (Gibson et al., 2017). A daily dose of prebiotics must be at least 2.5 gm (Costa et al., 2019) for its benefits. Prebiotic inulin not only promotes the growth of probiotic bacteria but also plays an important role in the technological properties of yogurt. The addition of 1% inulin enhances the sensory, textural, and microtextural properties of yogurt and improves the viability of yogurt bacteria (El-Kholy et al., 2020). Low-fat yogurt containing 0.5% (w/v) inulin improves the sensory properties and probiotic counts to 7.8 log cfu/g (Ghaderi-Ghahfarokhi et al., 2021). Many characteristics of prebiotic inulin make it highly compatible with yogurt fortification, such as solubility, degree of polymerization, fat replacer, stability at processing conditions (pH, temperature), and easily accessible to the probiotic yogurt bacteria (Kheto et al., 2023).

In India, mango fruits are easily available and could be used for the development of functional fruit yogurt. Mango fruit is rich in functional ingredients such as polyphenols, flavonoids, carotenoid pigment, vitamin C, and dietary fibers, which synergize with probiotic and prebiotic inulin to enhance products’ technofunctional properties and promote health benefits. Polyphenols and dietary fibers could have a significant prebiotic effect via promoting the growth of probiotic bacteria and other beneficial gut bacteria (Minj et al., 2024; Alves-Santos et al., 2020). These components can be metabolized by the probiotic and gut bacteria and provide them with a readily available energy source to exert various physiological functions such as inhibiting the growth of pathogenic bacteria, production of bioactive compounds, and immune modulatory components such as short-chain fatty acids (SCFA). These properties make probiotic bacteria flourish in the gut environment. Research studies have shown that the addition of mango juice in a fermented dairy-based beverage improves the viability of probiotic bacteria and improves the sensory properties (Ryan et al., 2020). In another study, supplementation with 1% *Moringa oleifera* leaf powder improves the overall technological properties, sensory attributes, and acceptability of mango-flavored yogurt (Saeed et al., 2021). Apart from technological properties, the potential health benefits of combining yogurt and fruits based on their prebiotic and probiotic properties are described by Fernandez and Marette (2017). Therefore, fortifying with mango fruit juice or pulp could have multiple benefits in the development of an innovative synbiotic mango fruit yogurt. A high-quality probiotic yogurt should have live probiotic bacteria, a smooth and firm texture, and good sensorial properties. Fortification with mango fruit juice or pulp will provide all the requirements in terms of product technological properties, prebiotic effects, health benefits, and overall acceptability.

Fortification with probiotic yogurt bacteria (Minj et al., 2020), prebiotic inulin (Minj and Vij, 2017), and mango fruit pulp in the development of synbiotic mango fruit yogurt will not only enhance its biofunctional and technological properties, but it will also be a great source of biologically active peptides. These bioactive peptides are generated by the proteolytic activity of yogurt bacteria during the fermentation process. These peptides are very specific protein fragments with a positive impact on human health. Many studies suggest that yogurt and derived bioactive peptides have various physiological roles, such as antioxidant and antimicrobial activities (Taha et al., 2017), ACE-inhibitory activity (Kim et al., 2021), antimutagenic activity (Heydari et al., 2023), antidiabetic and cytotoxic activity (Kayacan Çakmakoğlu et al., 2024), and immunomodulatory activity (Rivero-Pino et al., 2024). During yogurt production, the processing method and fermentation process are responsible for the release of small biologically active peptides from their native protein sequences. The size of biologically active peptides in milk and milk products can vary from 2 to 20 amino acids (Mohanty et al., 2016). The structural feature of a bioactive peptide is directly linked to its physiological function. For example, the peptide sequences VPYPQ, KVLPVPE (Rival et al., 2001), IPIQY, and GVRGPFPII (Hernández-Ledesma et al., 2005) from a yogurt sample have been reported for their radical scavenging activity whereas APFPEVFGK and FLPYPY (Chen et al., 2024) from peanut yogurt and HLPLP, IAK, and VYPFTGPIPN from milk-casein-derived peptide (Miguel et al., 2010) have been reported for ACE-inhibitory activity. The isolation and separation of these small molecular peptide sequences are possible with the application of ultrafiltration with molecular cutoff sizes of 3 kDa, 5 kDa, and 10 kDa. These sizes of bioactive peptide fractions have various biological activities, including antioxidant and ACE-inhibitory activities. Similarly sized peptide sequences from yogurt samples have been reported for their biological activities (Kayacan Çakmakoğlu et al., 2024; Karimi et al., 2021; Sabeena Farvin et al., 2010).

Fermented dairy product yogurt is made by yogurt bacteria, namely, *L. bulgaricus* subsp. *delbrueckii* and *S. thermophilus*. These lactic acid bacteria could produce proteolytic enzymes inside the cell (endopeptidases) as well as in the external medium (exopeptidases). These proteolytic enzymes break down the milk protein casein into smaller-size peptide sequences, which are responsible for specific biological activities such as antioxidant, antimicrobial, antidiabetic, anticancer, immune-modulatory, and ACE-inhibitory activities. Additionally, the use of well-proven probiotic yogurt bacteria, along with prebiotic inulin and mango fruit pulp, in the preparation of yogurt further increases its biofunctional properties. Mango fruit containing dietary fibers, polyphenols, and prebiotic inulin acts as a nutrient source for the probiotic yogurt bacteria and helps to maintain its viability and survivability in the gut for the long term. The addition of mango fruit pulp also increases its sensory properties. There is limited research available on the preparation of synbiotic mango fruit yogurt, along with its detailed biofunctional activities. A comprehensive study on the optimization of synbiotic mango yogurt preparation, chemical and technological analysis, isolation of bioactive peptides, and analysis of these bioactive peptides for biofunctional attributes could be an innovative idea for commercial synbiotic mango fruit yogurt production. Therefore, this study aims to optimize synbiotic mango fruit yogurt through response surface methodology, isolate the bioactive peptides, and analyze its biofunctional attributes, focusing on its antioxidant and ACE-inhibitory activities.

## 2 Materials and methods

### 2.1 Materials

Fresh, chilled, raw cow milk (∼3% fat) and skim milk (0.5% fat) were collected from the Experimental Dairy, ICAR-National Dairy Research Institute, Karnal, India. Spray-dried skim milk powder was collected from the Modern Dairy, Karnal, India. Sugar (Grade A) was obtained from the Experimental Dairy, ICAR-National Dairy Research Institute, Karnal, India. The frozen commercial mango fruit pulp (1 Kg pack) was procured in frozen and sterilized form from Delta Nutritive Mumbai. Mango pulp was thawed, mixed thoroughly, and aliquoted into individual packs of 90 g in sterile plastic bottles (Tarson) and stored at −20°C till use. Inulin as a prebiotic (Minj and Vij, 2017) was procured from Beneo Orafti, Mumbai, India. Polypropylene (PP) cups were procured from the Experimental Dairy, ICAR-National Dairy Research Institute, Karnal, India. Previously studied probiotic yogurt culture NCDC 144 (Minj et al., 2020) was used for the yogurt preparation.

### 2.2 Methods

#### 2.2.1 Preparation of synbiotic mango fruit yogurt

##### 2.2.1.1 Standardization

The fat percentage of cow milk was determined by the Gerber method (Kleyn et al., 2001). Standardization was done by adjusting the fat percentage of cow and skim milk using the Pearson square method (Tamime and Robinson, 1999). The solid-not-fat (SNF %) percentage of milk was calculated using the following formula and adjusted by adding skim milk.

$$\text{SNF }\left(\%\right)=\frac{\text{CLR}}{4}+\frac{F}{4}+0.44.$$

CLR: calibrated lactometer reading using an ISI lactometer at 29°C; F: fat (%)

##### 2.2.1.2 Synbiotic mango fruit yogurt preparation

**FIGURE 1:**
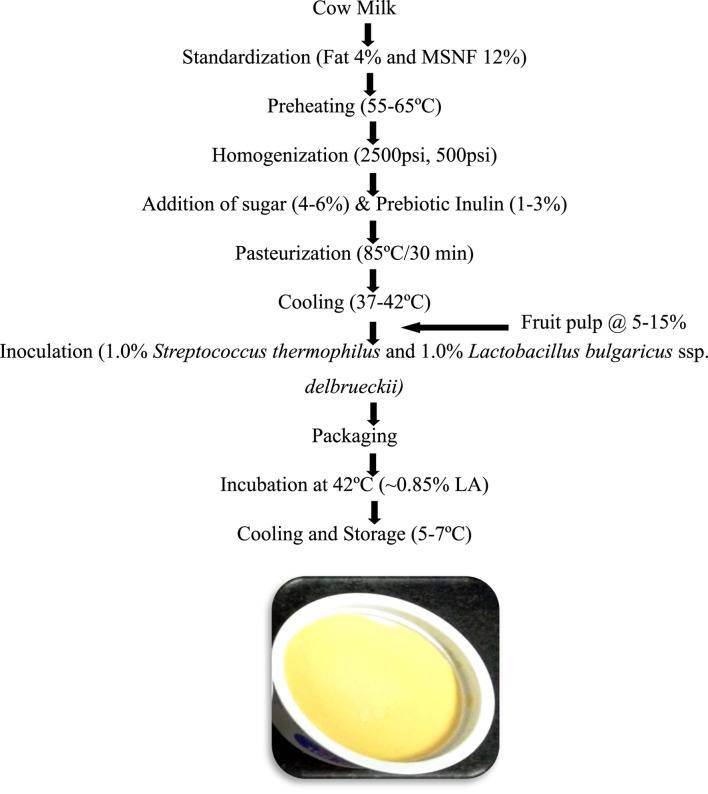
Flowchart for the preparation of synbiotic mango fruit yogurt. Probiotic yogurt cultures *Streptococcus thermophilus* 144 and *Lactobacillus bulgaricus* ssp. *delbrueckii* 144 (1:1) were used for the preparation of synbiotic mango fruit yogurt. MSNF, milk solids not fat; Fruit pulp, mango.

Synbiotic mango fruit yogurt was prepared as described in Figure 1.

#### 2.2.2 Optimization of independent factors for response antioxidant activity

Response surface methodology (RSM) was used for the optimization of three independent factors (sugar 4%–6%, prebiotic inulin 1%–3%, and mango fruit pulp 5%–15%) for the response antioxidant activity in the synbiotic mango fruit yogurt preparation. These independent factors were chosen based on our preliminary tests, where we found that very low and very high sugar and mango fruit pulp concentrations were not accepted by the consumers. In the case of prebiotic inulin, our previous research (Minj and Vij, 2017) demonstrated that 1%–3% supplementation of prebiotic inulin promotes the growth of probiotic bacteria. The values of independent factors at actual levels for 20 different runs were generated by the Design-Expert software for the dependent response antioxidant activity (Table 1).

**TABLE 1:** Experimental values of sugar, mango fruit pulp, and prebiotic inulin given by central composite rotatable design (CCRD).

Actual level			
Run	A	B	C
1	6	15	3
2	5	10	2
3	5	10	2
4	6	5	3
5	5	10	3.68179
6	5	10	2
7	5	18.409	2
8	4	15	3
9	3.31821	10	2
10	5	10	2
11	4	15	1
12	5	10	0.318207
13	5	10	2
14	4	5	3
15	6	5	1
16	4	5	1
17	5	1.59104	2
18	6.68179	10	2
19	5	10	2
20	6	15	1

Sugar % (A), fruit pulp % (B), and prebiotic inulin % (C).

Synbiotic mango fruit yogurt was prepared as per the actual levels (Table 1) to determine the response antioxidant activity.

##### 2.2.2.1 Determination of antioxidant activity by the ABTS method

This method assesses the total radical scavenging capacity based on the ability of a compound to scavenge the stable 2,2′-azinobis-(3-ethylbenzothiazoline-6-sulfonic acid) (ABTS) radical in 10 min (Re et al., 1999). The ABTS working solution was prepared and diluted with phosphate buffer saline (PBS) to adjust the absorbance at 734 nm to 0.7 ± 0.02. An aliquot of 10 µL of product supernatant, collected after centrifugation at 14,000×g for 30 min, was coated in a 96-well microplate, and to this, 100 µL ABTS in PBS solution was added and mixed for 10 s. The decrease in absorbance at 734 nm was recorded over a period of 10 min at 10 s intervals using a multi-plate reader. The results were expressed as Trolox equivalent antioxidant capacity (TEAC) values. A standard curve was prepared using Trolox concentrations of 0–1,000 µM.

#### 2.2.3 Preparation of synbiotic mango fruit yogurt extract and its bioactive peptides

Optimized synbiotic mango fruit yogurt extract was prepared by taking 10 g of sample in a 50 mL centrifuge tube and centrifuging it at 5,000 rpm for 5 min at 4°C. The supernatant was collected in another clean Falcon tube for analysis of the biofunctional properties.

##### 2.2.3.1 Extraction of bioactive peptides

The smaller size amino acid sequences of ∼2–20 amino acids are known for their biological activity.

Therefore, it is important to isolate these small molecular peptide sequences. Most of the small-size peptides were easily isolated and separated by ultrafiltration with molecular cutoff sizes of 3 kDa, 5 kDa, and 10 kDa. Therefore, the bioactive peptide fractions (10 kDa, 5 kDa, and 3 kDa) were extracted using a membrane cutoff Millipore filter. The filtrate was collected and analyzed for antioxidant activity by the ABTS method (described in Section 2.2.2.1) and angiotensin I (ACE)-inhibitory activity (described in Section 2.2.5.2). These methods have been previously adopted for the determination of antioxidant and ACE-inhibitory activity in isolated bioactive peptide samples (Singh et al., 2015).

#### 2.2.4 Physicochemical analysis of optimized synbiotic mango fruit yogurt

The optimized product has been analyzed for pH, acidity (Buono et al., 1990), total solids (TS) (AOAC, 2002), fat content by the Mojonnier extraction method (Richardson, 2001), and total protein content (Lowry et al., 1951).

#### 2.2.5 Biofunctional analysis of optimized synbiotic mango fruit yogurt

##### 2.2.5.1 Determination of antioxidant activity

Previously, the antioxidant activity of yogurt samples has been successfully determined and reported by the ABTS method (Pereira et al., 2024; Yoon et al., 2019), the 2, 2 diphenyl-1-picryl hydrazyl (DPPH) method (Atik et al., 2024; Yoon et al., 2019; Senadeera et al., 2018; Gjorgievski et al., 2014) and the ferric reducing antioxidant power (FRAP) method (Yoon et al., 2019; Senadeera et al., 2018). Therefore, we have adopted similar methods to determine the antioxidant activity in synbiotic mango fruit yogurt samples.

###### 2.2.5.1.1 Determination of antioxidant activity by the DPPH method

The antioxidant activity was determined by the DPPH method by following the method described by Brand-Williams et al. (1995) and Yoon et al. (2019) with some modifications. In brief, a stock solution was prepared by taking 27.7 mg of DPPH (Sigma–Aldrich, molecular weight-394.32) in a 50 mL amber reagent bottle and dissolving it in 25 mL methanol by stirring over a magnetic stirrer overnight at 4°C. The final volume was adjusted with methanol to 25 mL using a volumetric flask, and it was stored at −20°C. The working solution was prepared by dissolving 142 μL of stock in 9.858 mL of methanol. The working solution was prepared freshly prior to analysis and kept in an amber glass bottle. Next, 100 μL of the appropriate dilution of the product supernatant was loaded into a 96-well microplate and was mixed with 100 μL of freshly prepared DPPH working solution. The contents were mixed for 10 s and incubated in the dark for 120 min at 37°C after covering the microplate with aluminum foil. The absorbance of the solution was measured spectrophotometrically at 517 nm against methanol. For blank determination, 100 μL methanol was taken instead of the sample, and absorbance was measured immediately against methanol. The experiment was performed in triplicate.

The results were calculated and expressed as

$$\%\text{DPPH scavenging activity}=\frac{\left(A_{515}\;\text{nm}\;\text{blank}-A_{515}\;\text{nm}\;\text{sample}\right)}{A_{515}\text{nm}\;\text{blank}}x\;100$$

###### 2.2.5.1.2 Determination of antioxidant activity by the FRAP method

The ferric-reducing antioxidant potential assay was conducted following the method described by Benzie and Strain (1996) with some modifications. In brief, a stock solution of acetate buffer (300 mM, pH 3.6 ) was made by mixing 3.1 g of sodium acetate trihydrate (molecular weight 136.08) in 16 mL of glacial acetic acid, and the final volume was made up to 1,000 mL with distilled water. The FRAP reagent was prepared by mixing 10 mL of 300 mM acetate buffer ( pH 3.6 ), 1 mL of 10 mM TPTZ (molecular weight 312.34) solution (10 mM TPTZ in 40 mM HCl), and 1 mL of 20 mM FeCl_3_ (molecular weight 270.30) solution (i.e., ratio 10:1:1 v/v). Fresh reagent was prepared immediately before analysis. Next, 10 μL of product supernatant was loaded into a 96-well microplate and mixed with 100 μL of FRAP reagent. The samples were mixed and then incubated at 37°C for 30 min. The increase in absorbance was measured by a spectrophotometer at 593 nm against the acetate buffer. For reagent blank preparation, 10 μL of distilled water was taken and subtracted from the sample reading to calculate the increase in absorbance for each sample. The results were expressed as ferrous sulfate equivalent values. The experiment was performed in triplicate. The standard curve of FeSO_4_ was prepared by a stock solution of 2 mM concentration that was diluted to 100–1,000 μM with distilled water.

##### 2.2.5.2 Angiotensin I (ACE) inhibitory activity

ACE inhibitory activity was determined following the method described by Cushman and Cheung (1971). In brief, approximately 25°g of the product was centrifuged at 4,000°g for 15 min at 4°C. The supernatant was collected, and the pH was subsequently adjusted to 8.3 using 10 M NaOH. The suspension was centrifuged for 5 min at 14,000×g at 4°C. Thereafter, 110 µL of 5 mM HHL solution was mixed with 100 µL of 0.1 M sodium borate buffer ( pH 8.3 ) and 20 µL of sample was added. The reaction was initiated by the addition of 20 µL (4 mU in 250 µL) of ACE enzyme, and the mixture was incubated for 30 min at 37°C. The reaction was terminated by the addition of 250 µL of 1 N HCl. The hippuric acid liberated by the ACE was extracted with 1.5 mL ethyl acetate by centrifugation at 3,000 g for 10 min and heat evaporated at 95°C for 10 min. The residue containing hippuric acid was dissolved in 1 mL of deionized water, and the absorbance of the solution was measured spectrophotometrically using a Spectro UV-VIS Double Beam Research Spectrophotometer (UV-VIS Double Beam Model- UVD-3500, United States) at 228 nm against a blank. The blank included all components except ACE, and for the control, distilled water was added in place of the sample. The extent of inhibition was calculated as follows:

$$\frac{\left(B-A\right)}{\left(B-C\right)}x\;100$$

where

A = the absorbance in the presence of ACE and the ACE-inhibitory component (sample),
B = the absorbance without the ACE-inhibitory component (control) and
C = the absorbance without ACE (blank).

Inhibition was expressed as the concentration of component that inhibits 50% of ACE activity (IC_50_), and 1 unit of ACE-inhibitory activity was expressed as the potency showing 50% ACE inhibition under these conditions.

##### 2.2.5.3 Determination of proteolytic activity

The proteolytic activity of optimized synbiotic mango fruit yogurt was determined by measuring liberated amino acids and peptides using the o-phthalaldehyde (OPA) method described by Church et al. (1983) with some modifications. In brief, 2.5 mL of sample supernatant was added to 5 mL of 0.75% trichloroacetic acid and allowed to stand for 10 min, and the mixture was filtered using Whatman filter paper 42 (Quantitative Filter Paper, Ashless, Whatman 1,442-055, United States). Then 150 µL of permeate was added to 3 mL of OPA reagent, which was kept on ice. After 2 min of incubation at room temperature (20^o^C), the absorbance of the solution was measured spectrophotometrically at 340 nm. The standard curve was prepared using serine with a concentration range of 25–250 μg/mL. The proteolytic activity was expressed as serine equivalents.

##### 2.2.5.4 Total polyphenol content

The total phenolic content of synbiotic mango fruit yogurt was analyzed using the Folin–Ciocalteu method (Kähkönen et al., 1999). In brief, 400 μL of appropriately diluted sample/gallic acid standard was taken in a test tube. Next, 2000 μL of diluted Folin–Ciocalteu reagent was added and mixed on a vortex mixer (Mohammed et al., 2019). After 3 min, 1,600 μL of sodium carbonate solution was added and incubated under dark at room temperature for 30 min. For the blank preparation, 400 μL of distilled water was taken instead of the sample. The absorbance of the sample was measured against a blank at 765 nm by a double-beam spectrophotometer (Shimadzu, Japan). The standard curve was prepared with 400 μL of a 10–100 μg/mL concentration of the gallic acid solution.

##### 2.2.5.5 Total flavonoid content

Total flavonoid contents were determined by following the method described by Zhishen et al. (1999). In brief, a 0.5 mL volume of 2% AlCl_3_ ethanol solution was added to 0.5 mL of sample solution. After 1 h at room temperature, the absorbance was measured at 420 nm using a double-beam spectrophotometer (Shimadzu, Japan). A yellow color indicated the presence of flavonoids. Distilled water was used as a blank and a control. Extracted samples were evaluated at a final concentration of 0.5 mg/mL. The results were reported as quercetin equivalents per mL of extract. The standard curve was prepared with 0.5 mL of a 1–6 mg/100 mL concentration of quercetin.

#### 2.2.6 Technological analysis of optimized synbiotic mango fruit yogurt

##### 2.2.6.1 Texture profile analysis

The texture profile of synbiotic mango fruit yogurt was determined following the method described by Narender Raju and Pal (2009) using a texture analyzer TAXT21 (Stable Micro Systems, Godalming, Surrey, United Kingdom) fitted with a 5 kg load cell. For texture analysis, samples were prepared in 100 mL sterilized sample containers, and the set samples were kept in an immersion chamber maintained at 25°C before the analysis. The samples were subjected to mono-axial compression of 20 mm distance on the texture analyzer by the crosshead speed of 2 mm/s.

The test conditions maintained were as follows:

Measured force in compression
Probe P25 cylindrical (diameter 25 mm)
Load cell = 25 kg
Pre-test speed = 2 mm/s
Test speed = 1 mm/s
Post-test speed = 2 mm/s
Compression = 50% of distance
Single TPA (texture profile analysis) = single-time penetration

After completion of the analysis, a graph was obtained with the force experienced by the probe on the *Y*-axis and time on the *X*-axis. The firmness of the yogurt sample was estimated as the height of the positive peak force up to the rupture point.

##### 2.2.6.2 Flow behavior

The flow behavior property of synbiotic mango fruit yogurt was analyzed following the method described by Meena et al. (2016). In brief, a rheometer (Modular compact rheometer, MCR 52) attached with a cone and plate geometry (CP 75) with 0.149 mm gap setting at 20°C constant temperature using a variable shear rate (0–100 s^-1^) was used to measure the flow behavior.

##### 2.2.6.3 Water holding capacity (WHC)

The water-holding capacity was determined by following the method described by Remeuf et al. (2003). In brief, 20 g of optimized synbiotic mango fruit yogurt was taken in 50 mL centrifuge tubes and centrifuged at 2000 rpm for 10 min. The weight of separated clear whey was measured, and whey syneresis was expressed as grams of whey per 20 g of sample. The water holding capacity (WHC) % was expressed as

$$\text{WHC }\left(\%\right)=100\times \frac{\text{NY}-\text{WE}}{\text{NY}}$$

where NY = Weight of native synbiotic mango fruit yogurt
WE = Weight of expelled whey

#### 2.2.7 Microbiological quality analysis

Microbiological quality analysis for total count and coliform count was performed according to the method described by Arnott et al. (1974) for the milk quality as well as for the optimized product quality.

#### 2.2.8 Statistical analysis

The response surface methodology (RSM) was adopted for the experimental design and analysis. Multiple regression analysis was done to fit the model. Statistical analyses of the data and 2D-contour and 3D plot designs were computed using Design-Expert software (V 23.1.4; Stat-Ease Corporation, Minneapolis, MN, United States). Other experimental data were analyzed in GraphPad Prism version 10.4.0 (527) software.

## 3 Results and discussion

Previously studied prebiotic inulin (Minj and Vij, 2017) and probiotic yogurt culture NCDC 144 (Minj et al., 2020) were used for the preparation of synbiotic mango fruit yogurt. Raw materials such as cow milk and mango fruit pulp were analyzed before the preparation of the synbiotic mango fruit yogurt. Cow milk was analyzed for fat and SNF content and microbiological quality. As per the requirements, the fat content was adjusted to 3.0% either by cream separation or by adding extra cream. Adjustment of fat content is important because it has a significant impact on the quality of yogurt, such as viscosity, taste, appearance, texture, flavor, and aroma. Milk fat breakdown is the main source for the accumulation of free fatty acids, which could be a precursor of aroma compounds in the yogurt. Milk fat also helps to reduce whey separation by enhancing the water-holding capacity and increasing the adhesiveness and hardness (Su et al., 2022). Similarly, SNF content is important in yogurt because it affects the viscosity, firmness, cohesiveness, consistency, fermentation, and coagulation time. SNF content increases the cohesiveness and firmness of yogurt (Walstra et al., 2006); positively affects viscoelasticity, texture, and viscosity of the yogurt (Yu et al., 2016); and increases the duration of fermentation time (Kristo et al., 2003). In this study, the SNF content was adjusted to 11%–12% by the addition of skimmed milk powder (SMP). The whole standardization procedure was followed by the Pearson square method. Results of standardized fat and SNF contents and microbiological quality of cow milk are presented in Table 2.

**TABLE 2:** Fat, SNF, and microbiological quality analysis of standardized milk.

Milk	Fat	SNF	Total count	Coliform count
Cow milk	3.03 ± 0.03	12.03 ± 0.07	ND	ND

Data are in mean ± SEM (3 replicates); not detected (ND).

Similarly, mango fruit pulp was also analyzed for the pH, acidity, and microbiological quality. The *pH* and acidity of mango fruit pulp were *fruit pulp were* 4.35 ± 0.01 and 1.25% ± 0.01%, respectively (Table 3). The *pH* value of fresh mango pulp was reported slightly lower *was reported slightly lower*, at 4.21 ± 0.0, by Laophongphit et al. (2024) and 4.02 ± 0.15 by Kaushik et al. (2014), whereas the acidity of mango pulp was reported as 0.48% ± 0.05% TA (lactic acid) and 0.49 ± 0.09 g citric acid/100 g pulp, respectively, which was quite lower than our results.

**TABLE 3:** pH, acidity, and microbiological quality analysis of mango fruit pulp.

Sample	pH	Acidity (%citric acid)	Total count	Coliform count
Mango fruit pulp	4.35 ± 0.01	1.25 ± 0.01	ND	ND

Data are in mean ± SEM (3 replicates); not detected (ND).

### 3.1 Optimization of process conditions for the preparation of synbiotic mango fruit yogurt

There is an increasing trend of dairy product formulation, mainly yogurt, to enhance its functional, nutritional, textural, and technological properties. Fortification with any form of fruit (raw fruit, pulp, dried form) not only enhances its palatability and appearance but also improves the overall quality of the product. Because fruits are rich in minerals, polyphenols, flavonoids, fibers, and carotenoids, they significantly affect the overall quality of fortified products. Many health benefits of mango fruit and fruit pulp intake have been reported, including antioxidant, anti-inflammatory, and anticancer properties (Lauricella et al., 2017). Prebiotic inulin, on the other hand, also improves the physicochemical properties of yogurt and promotes the growth of probiotic bacteria (Minj and Vij, 2017; Minj et al., 2020). A high-quality yogurt should have a high consumer acceptance score in terms of flavor and taste. Therefore, the addition of sugar is also important to increase the overall acceptability of synbiotic mango fruit yogurt. Overall, the present study was conducted to optimize the fruit pulp, prebiotic inulin, and sugar content to develop a high-quality biofunctional rich yogurt by focusing on its antioxidant and ACE-inhibitory activities.

The 20 different runs of the combined effect of sugar (A), fruit pulp (B), and prebiotic inulin (C) on the antioxidant activity of synbiotic mango fruit yogurt are shown in Table 4. The results showed that the lowest antioxidant activity was 268.336 μmol/L TEAC, and the second lowest antioxidant activity was 665.872 μmol/L TEAC. These results indicated that the low concentration of mango fruit pulp fortification significantly affected the antioxidant activity of the synbiotic mango fruit yogurt. According to the reports, the fortification of mango increases the antioxidant activity of probiotic yogurt compared to the control yogurt (Mohamed et al., 2023). Overall, the antioxidant activity of synbiotic mango fruit yogurt ranged from 268.336 µmolL/ TEACH to 3,972.04 μmol/L TEAC.

**TABLE 4:** Response value of the antioxidant activity of synbiotic mango fruit yogurt.

	Independent factors (actual value)			Response
Run	A	B	C	Antioxidant activity (μmol/L TEAC)
1	5	10	2	3,972.04
2	4	5	3	1,669.65
3	5	10	2	1,778.97
4	5	10	3.68179	2,418.34
5	5	10	0.318207	1,600.08
6	4	15	3	2,696.61
7	5	10	2	3,972.04
8	6	5	1	665.872
9	5	10	2	3,972.04
10	6	15	3	3,660.64
11	6	5	3	1,570.26
12	5	10	2	3,972.04
13	4	15	1	2,116.88
14	4	5	1	2,733.06
15	5	10	2	3,972.04
16	6	15	1	2,073.81
17	5	18.409	2	2,355.4
18	5	1.59104	2	268.336
19	3.31821	10	2	3,972.04
20	6.68179	10	2	2,097.00

Antioxidant activity was determined using the ABTS method.

TEAC: Trolox equivalent antioxidant capacity (μmol/liters).

Sugar % (A), fruit pulp % (B), and prebiotic inulin % (C).

#### 3.1.1 Diagnostic check of the fitted model

The experimental data of the central composite rotatable design (CCRD) were fitted and calculated with a second-order quadratic function and multiple linear regressions. The model adequacy was checked using the coefficient of correlation, lack of fit test, and F-ratio (Table 5). The correlation coefficient was 0.89. The lack of fit test was found to be insignificant, which indicates that the model is adequately accurate to predict the antioxidant activity for any combination of independent factors in the ranges studied. This suggested that the obtained model can be used to determine the relative effect of the independent factors, determine the optimum parameter combinations for the highest and desirable response antioxidant activity, and predict the result.

**TABLE 5:** Regression coefficients of independent coded factors and ANOVA data for the response (antioxidant activity in µmol/L TEAC) in synbiotic mango fruit yogurt.

Coefficient	Antioxidant activity µmol/L TEAC
Intercept	1.606.29
A	14.02
B	466.86**
C	0.49
AB	−30.64
AC	−2.48
BC	8.28
A^2^	−32.12
B^2^	−95.37
C^2^	−35.64
Model (*p*-value)	0.0011**
Lack of Fit (*p*-value)	0.0566
R^2^	0.8883

*Significant at *p* ≤ 0.05; ** Significant at *p* ≤ 0.01

Antioxidant activity was determined using the ABTS method.

TEAC, Trolox equivalent antioxidant capacity (μmol/L).

A, B, and C are regression coefficients for sugar, mango fruit pulp, and prebiotic inulin, respectively.

#### 3.1.2 RSM model for antioxidant activity

The quadratic model fitted to antioxidant activity was significant (*p* ≤ 0.05). Lack of fit was insignificant relative to pure error.

$$Y=-1023.77588+401.51165\;*\;A+196.99947\;*\;B+138.87961\;*\;C-6.12875\;*\;A\;*\;B-2.48375\;*\;A\;*\;C+1.65675\;*\;B\;*\;C-32.12317\;*\;A^{2}-3.81484\;*\;B^{2}-35.63572\;*\;C^{2}$$

where Y represents the response antioxidant activity, and test variables are A (sugar), B (mango fruit pulp), and C (prebiotic inulin).

All three variables individually affected the antioxidant activity. The antioxidant activity was minimal at a low concentration of mango pulp and increased with increased concentration except for the very high concentration. Interestingly, sugar concentration and prebiotic inulin concentration have less impact on the antioxidant activity of the synbiotic mango fruit yogurt.

#### 3.1.3 Optimization of the level of independent factors

In order to optimize the level of independent factors, the response antioxidant activity was assigned equal importance on the basis of their effect on biofunctional properties as well as the overall acceptability of the synbiotic mango fruit yogurt. The criterion used and predicted and actual responses are given in Table 6. With the model, the optimized value for sugar concentration was 6%, the value for mango fruit pulp was 6.562%, and the value for prebiotic inulin was 1%. The synbiotic mango fruit yogurt was developed according to the optimized process conditions. The actual experiment value for antioxidant activity was found to be very close to the predicted value (Table 6). The t-test analysis revealed that there were no significant ( p>0.05 ) changes in the predicted and experimental value of antioxidant activity.

**TABLE 6:** Predicted and experimental response values at the optimum formulation condition and t-test value.

Factor	Response (antioxidant activity) μmol/L TEAC				t-test value
Sugar (%)	Mango fruit pulp (%)	Prebiotic inulin (%)	Predicted value	Experimental value	0.53 (*p* > 0.05)
6.00%	6.562%	1.00%	1,214.177	1,213.586	

*p* > 0.05 indicates no significant difference.

Antioxidant activity was determined using the ABTS method.

TEAC, Trolox equivalent antioxidant capacity (μmol/L).

**FIGURE 2:**
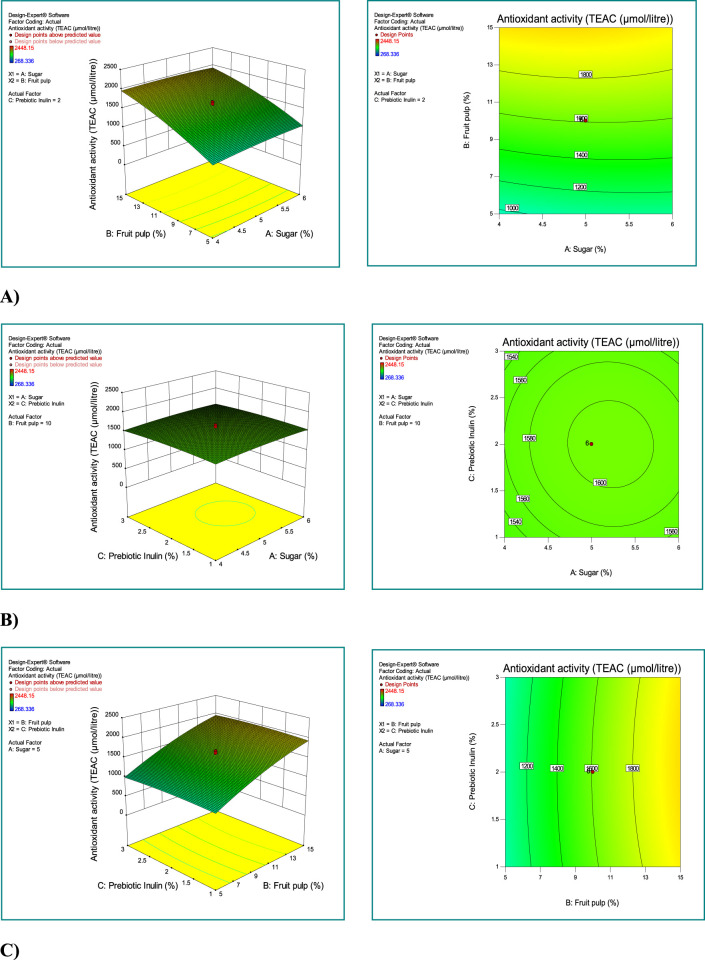
Investigating the simultaneous effect of independent variables (sugar, fruit pulp, and prebiotic inulin) on the response antioxidant activity. **(A)** Combined effect of fruit pulp and sugar concentration on antioxidant activity; **(B)** combined effect of prebiotic inulin and sugar concentration on antioxidant activity; **(C)** combined effect of prebiotic inulin and mango fruit pulp concentration on antioxidant activity. Response surface plots for antioxidant activity; 3D plots (left side) and 2D contour plots (right side).

The interaction between sugar, mango fruit pulp, and prebiotic inulin is depicted in Figure 2. As the concentration of mango fruit pulp increased, the antioxidant activity also increased (Figure 2A; 3D-plot and 2D-contour). The interaction between prebiotic inulin and sugar concentration slightly increased the antioxidant activity of the synbiotic mango fruit yogurt (Figure 2B; 3D-plot and 2D-contour). The third interactive graph 3D-plot and 2D-contour) between the prebiotic inulin and mango fruit pulp showed that the antioxidant activity was not affected by the combination of these two factors (Figure 2C).

**FIGURE 3:**
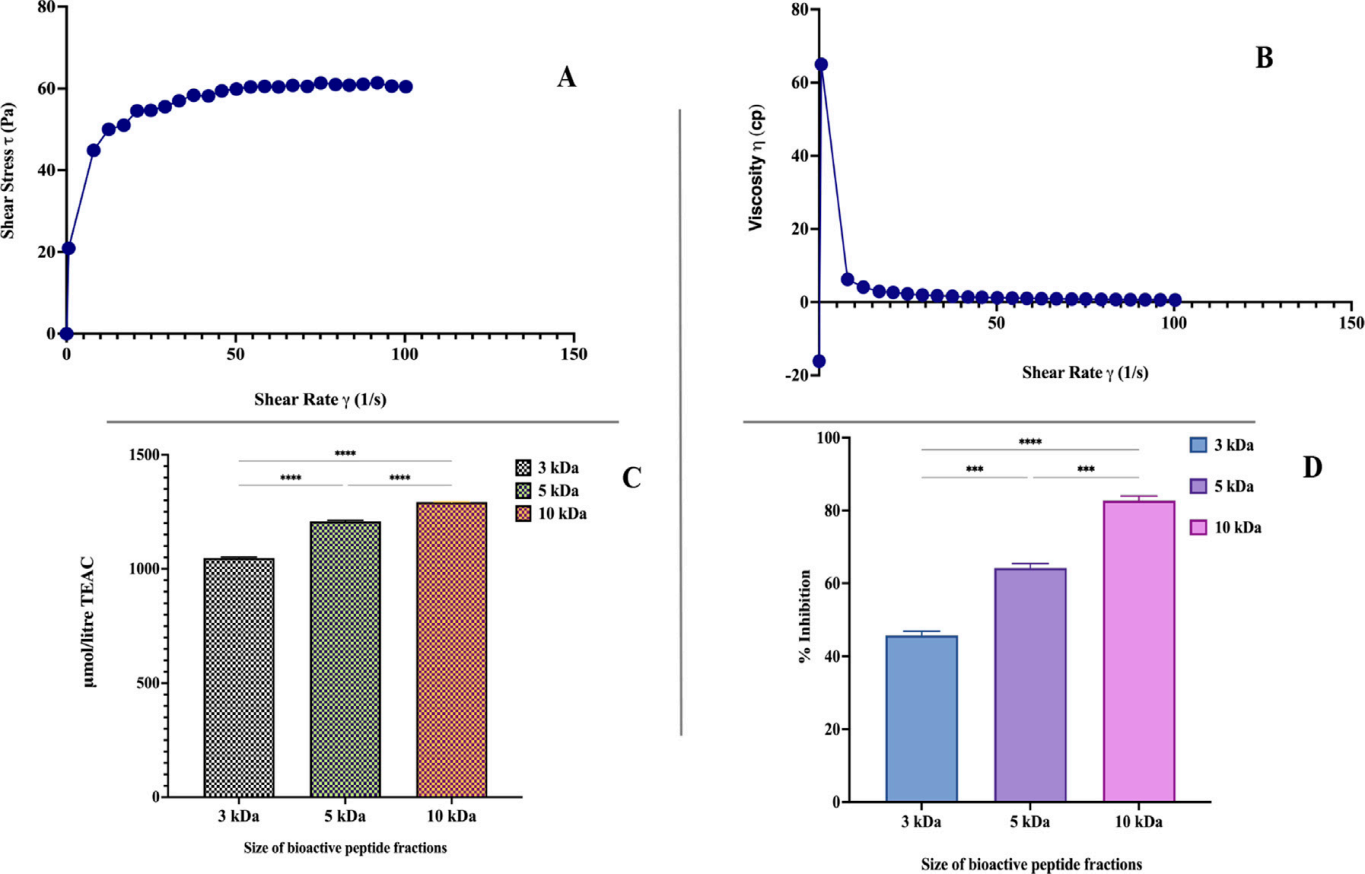
**(A)** Shear stress as a function of the flow behavior of synbiotic mango fruit yogurt. **(B)** Relationship between apparent viscosity and shear rate of synbiotic mango fruit yogurt. **(C)** Antioxidant activity of synbiotic mango fruit yogurt derived-bioactive peptide fractions (3 kDa, 5 kDa, and 10 kDa). **(D)** ACE-inhibitory activity of synbiotic mango fruit yogurt-derived bioactive peptide fractions (3 kDa, 5 kDa, and 10 kDa). * indicates results differ significantly ( p<0.0001 ). Antioxidant activity was determined using the ABTS method in triplicate. TEAC, Trolox equivalent antioxidant capacity μmol/L. ACE inhibitory activity was determined in triplicate.

### 3.2 Physicochemical and biofunctional analysis of optimized synbiotic mango fruit yogurt

Synbiotic mango fruit yogurt was prepared under optimized conditions as per the values given in Table 6, and the physicochemical and biofunctional properties were analyzed. The physicochemical parameters such as fat, TS, protein content, pH, acidity, WHC, polyphenol content, proteolytic activity, and flavonoid content were determined and are shown in Table 7. The fat content of 3.07% ± 0.19%, TS content of 22.91% ± 1.15%, and protein content of 0.39 ± 0.00 mg/mL were found in the optimized synbiotic mango fruit yogurt. The pH and acidity values were 4.65 ± 0.01 and 0.65% ± 0.00% lactic acid, respectively. The WHC of synbiotic mango fruit yogurt was determined to be 93.67% ± 0.33%.

**TABLE 7:** Physicochemical and biofunctional properties of optimized synbiotic mango fruit yogurt.

Composition/properties	Synbiotic mango fruit yogurt
Fat (%)	3.07 ± 0.19
Total solids (TS) (%)	22.91 ± 1.15
Protein (mg/mL)	0.39 ± 0.00
pH	4.65 ± 0.01
Acidity (% lactic acid)	0.65 ± 0.00
Water holding capacity (%)	93.67 ± 0.33
Polyphenol content (mg GAE/100 mL)	270 ± 0.60
Proteolytic activity (μg serine /mL)	198.61 ± 0.50
Flavonoid content (quercetin equivalent/mL)	0.86 ± 0.00
ACE-inhibitory activity (% inhibition)	58.15 ± 0.08
Antioxidant activity by different methods	
ABTS (μmol/liter TEAC)	1213.59 ± 1.10
FRAP (FeSo_4_ μmol/mL)	809.87 ± 1.86
DPPH (% inhibition)	44.52 ± 0.28

GAE: mg gallic acid equivalent/100 mL.

TEAC, Trolox equivalent antioxidant capacity (μmol/L).

Data are in mean ± SEM (n = 3).

ACE-inhibitory activity, angiotensin-converting enzyme I-inhibitory activity; ABTS, 2,2′-azinobis-(3-ethylbenzothiazoline-6-sulfonic acid); FRAP, ferric reducing antioxidant power; DPPH, 2, 2 diphenyl-1-picryl hydrazyl.

The biofunctional properties of plain yogurt could be enhanced by fortification with fruits, prebiotics, probiotics, and proteolytic bacteria. Fortification with fruit pulp not only enhances the sensory properties but also enhances potential antioxidant compounds such as total phenolic compounds, flavonoid content, and prebiotic components, which further increases the viability of probiotic bacteria. In this study, the polyphenol and flavonoid contents in synbiotic mango fruit yogurt were noted as 270 ± 0.60 mg GAE/100 mL and 0.86 ± 0.00 quercetin equivalent/mL, respectively. Fortification with different fruit pulps has different impacts on the functionality of yogurt. Pereira et al. (2024) used a similar Folin–Ciocalteau method to evaluate the total phenols of different types of exotic fruit pulp-fortified yogurt and reported that yogurt containing araçá, blueberry, and grumixama had a significant increase of total phenolics after the fermentation process, but it decreased at the end of the storage at 28 days. Pădureţ et al. (2024) reported that yogurts supplemented with black chokeberry fruit, juice, and pomace have different levels of total phenolic content with the highest content in yogurt with chokeberry pomace at 3% (392.14 ± 2.06 µg GAE/g) followed by the lowest in yogurt sample with 1% chokeberry juice (104.45 ± 2.63 µg GAE/g). In another study, Zahid et al. (2023) reported that mango peel powder improves the prebiotic functions and health-promoting effect of fortified yogurt. The presence of phenolic compounds improved the production of SCFA and the microbial population associated with yogurt fortified with mango peel powder and lactic acid bacteria. Therefore, fortification with different fruit components could be a promising method of developing biofunctional fruit yogurt to increase its commercial value and effect on gut health.

The incorporation of probiotic bacteria and prebiotics, such as inulin, improves the proteolytic activity of yogurt. Pinto et al. (2020) reported that the degree of hydrolysis of yogurt B (yogurt starter + probiotic *L. acidophilus* + *Bifidobacteria* spp.) was almost double that of yogurt A (conventional yogurt added only yogurt starter). The degree of hydrolysis of yogurt C (yogurt starter + probiotic *L. acidophilus* + *Bifidobacteria* spp. + 1% inulin (w/w)) appeared to be more pronounced than yogurt B. The higher concentration of prebiotic inulin in yogurt D (yogurt starter + probiotic *L. acidophilus* + *Bifidobacteria* spp. + 3% inulin (w/w)) greatly modified the proteolytic activity. Therefore, fortifying yogurt with probiotics, prebiotics, and mango fruits containing dietary fibers could increase its proteolytic activity. In this study, the synbiotic mango fruit yogurt showed 198.61 ± 0.50 μg serine/mL proteolytic activity.

The ACE-inhibitory activity of synbiotic mango fruit yogurt was estimated as 58.15% ± 0.08% inhibition. Wang et al. (2024) reported that fortification of yogurt with *Lactiplantibacillus plantarum* and casein significantly affects the ACE-inhibitory activity of yogurt. Yogurt group 883 + M11-CS fortified with starter culture YO-MIX 883 (0.5 × 10^7^), *Lb. plantarum* M11 (1 × 10^7^), and sodium caseinate (2%) had a significantly higher ACE inhibition rate of 83.15% ± 0.52% than yogurt group 883-CS, which was fortified with YO-MIX 883 (1.5 × 10^7^) and sodium caseinate (2%), which had a 78.39% ± 0.56% ACE inhibition rate. Yogurt group 883 + M11, which was fortified with YO-MIX 883 (0.5 × 10^7^) and *Lb. plantarum* M11 (1 × 10^7^), showed a 77.65% ± 0.51% ACE inhibition rate. Yogurt group 883 fortified with YO-MIX 883 (1.5 × 10^7^) exhibited the lowest ACE inhibition rate, at 74.73% ± 0.89%. The ACE-inhibitory activity of the synbiotic mango fruit yogurt was comparatively less than this report because different components affect the ACE inhibition rate of yogurt. Supplementation of probiotic culture along with yogurt culture also improves the ACE-inhibitory activity of yogurt. Kim et al. (2021) reported that yogurt supplemented with *Lactiplantibacillus plantarum* NK181 had the highest ACE-inhibitory activity (51.3% ± 10.3%) of other supplemented yogurt samples. The ACE-inhibitory activity was comparatively higher in this study in synbiotic mango fruit yogurt. Yi et al. (2024) reported that fermented buffalo milk yogurt with commercial starter and probiotic *Lactobacillus plantarum* B exhibited 84.51% ACE-inhibitory activity that showed an antihypertensive effect in the pregnancy-induced hypertensive rat. An *in vivo* study on the antihypertensive effect of synbiotic mango fruit yogurt is needed in the future.

The antioxidant activities of synbiotic mango fruit yogurt were 1,213.59 ± 1.10 μmol/L TEAC, 809.87 ± 1.86 FeSo4 μmol/mL, and 44.52% ± 0.28% inhibition by the ABTS, FRAP, and DPPH methods, respectively. Pădureţ et al. (2024) reported that yogurts supplemented with different concentrations of black chokeberry fruit, juice, and pomace had different DPPH radical scavenging activities. Yogurt supplemented with 3% chokeberry fruit had higher inhibition of DPPH radical formation, followed by 2% chokeberry fruit supplementation (73.57% ± 0.11%) and 1% chokeberry fruit supplementation (57.84% ± 0.05%); yogurt with 3% chokeberry fruit pomace had up to 64.8% ± 0.11% inhibition. According to this study, adding fruit and fruit components increased the antioxidant activity of yogurt, which supports our results.

According to Erkaya-Kotan (2020), the percent DPPH scavenging is a criterion for *in vitro* antioxidant activity. The antioxidant activity of probiotic yogurt incorporated with orange fiber increased during 14 days of storage, while it decreased until the 21st day of storage. Yogurt incorporated with orange fiber had significantly higher antioxidant activity (23.56%–32.94%) than the control sample (20.36%–24.51%) during all days of storage. In another study, the antioxidant activity of a water-soluble peptide extract (WSPE) of fresh and cold stored yogurt samples was determined by ABTS and DPPH assays. The WSPE of fresh and cold stored yogurt samples made with yogurt culture alone had the highest (13.7%) antioxidant activity by ABTS assay compared to the WSPE of *Lb. acidophilus* 20,552 ATCC + yogurt culture (13.2%) and the WSPE of yogurt culture along with *Lb. helveticus* CH5 (10.5%). Similarly, the DPPH scavenging activity of the same samples was 56.88%–71.71%, 70.14%–80.62%, and 79.45%–81.62%, respectively (Taha et al., 2017). Although the antioxidant activity of synbiotic mango fruit yogurt extract by DPPH (44.52% ± 0.28%) was less than this research study, it was higher than the antioxidant activity of fermented milk (39.43%) made by the symbiotic cultures and *Lactobacillus delbrueckii* ssp. *bulgaricus* and *S. thermophilus* (Gjorgievski et al., 2014). Yogurt culture *L. delbrueckii* ssp. *bulgaricus* could ferment milk protein into amino acid sequences that affect radical scavenging activity (Kudoh et al., 2001). Kim et al. (2021) also reported that yogurt supplemented with *L. delbrueckii* KU200171 had the highest antioxidant activities by ABTS (39.3% ± 1.0%) and DPPH assays (86.5% ± 0.3%) compared to other probiotic culture-supplemented yogurt samples. Fruit supplementation improves the antioxidant activity of yogurt, and supplementation with prebiotic and lactic acid bacteria also improves oxidative stress in adults with metabolic syndrome (Zolghadrpour et al., 2024).

Many fermented dairy products have been studied for their potential antioxidant activity due to the presence of antioxidant peptide sequences. Recently, the antioxidant activity of probiotic fermented milk cheese ultrafiltered fractions and water-soluble extracts have been reported for their potential antioxidant activity. Peptides AMKPWIQPK and EMPFPK exhibited antioxidant properties due to their amino acid sequences, such as methionine and proline residues (Yang, 2024). The presence of lactic acid bacteria in fermented milk is important in the production of antioxidant peptides. The lactic acid bacteria isolated from Iranian dairy products have the potential for antioxidant activity. The isolated *L. delbrueckii* PTCC 1900 had the highest (59.43%) DPPH free radical scavenging activity, and *L. paracasei* ssp. *paracasei* PTCC 1945 had the highest (85.93%) ABTS free radical scavenging activity at 48 h (Shirkhan et al., 2023).

### 3.3 Technological analysis of synbiotic mango fruit yogurt

Texture profile analysis of optimized synbiotic mango fruit yogurt was done on the basis of a back extrusion test, which was suited for the gels because it is not affected by the free whey on the surface of the samples (Pereira et al., 2003). For this, different parameters, such as firmness, work of shear, work of adhesion, and stickiness, were taken into consideration. The firmness value is the peak force obtained during penetration of the probe in the synbiotic mango fruit yogurt (Table 8). The values for firmness, work of adhesion, work of shear, and stickiness were 1.70 ± 0.06 N, −4.58 ± 0.44 N s, 32.78 ± 2.73 N s, and −0.48 ± 0.04 N, respectively (Table 8). The flow behavior and viscosity of optimized synbiotic mango fruit yogurt are presented in Figures 3A, B, respectively.

**TABLE 8:** Textural analysis of synbiotic mango fruit yogurt.

Parameters	Value
Firmness N	1.70 ± 0.06
Work of adhesion N s	−4.58 ± 0.44
Work of shear N s	32.78 ± 2.73
Stickiness N	−0.48 ± 0.04

The improvements in textural profile and viscosity could be due to the addition of prebiotic inulin and mango fruit pulp containing dietary fibers. Brennan and Tudorica (2008) reported that levels above 2% w/w of inulin are needed to exert significant improvements in apparent viscosity. In this study, the optimized condition for prebiotic inulin was 1%. This concentration of inulin was sufficient to maintain the viscosity and textural properties of the product because mango fruit pulp also contains some amounts of dietary fiber. According to Buriti et al. (2008), inulin is responsible for the apparent viscosity and firmness in the symbiotic cheese. The addition of passion fruit peel powder and vegetal oil emulsion did not influence the fermentation time, but the firmness of the yogurt was significantly affected (Perina et al., 2015).

The value of synbiotic mango fruit yogurt increases with its beneficial health aspects, technological properties, and consumer acceptance. Janoušek Honesová et al. (2024) reported that yogurt made with the addition of skimmed milk powder and fruit jams improved the acceptance of fruit yogurt compared to natural yogurt. Supplementations with different fruits have different acceptance rates. Pădureţ et al. (2024) reported that yogurt incorporated with 3% and 2% black chokeberry had the highest acceptance score, while a yogurt sample with 3% black chokeberry pomace had the lowest acceptance score. Saeed et al. (2021) studied the physicochemical properties of mango-flavored yogurt supplemented with moringa oleifera leaf powder and reported that supplementation with 1% moringa oleifera leaf powder had the highest sensory attribute scores and overall acceptability. Recently, Cao et al. (2024) reported that yogurt incorporated with red beetroot powder (3%) showed the highest viable counts, followed by the second highest viable counts in mango fruit powder (3%)-fortified yogurt after 15 days of storage at refrigerated storage. Yogurt fortified with mango fruit powder had the highest consumer acceptability scores for texture, flavor, and overall acceptability, while other yogurts had lower acceptability scores. The initial study on the development of synbiotic mango fruit yogurt also scored the highest sensory attributes and overall acceptance.

### 3.4 Determination of the biofunctional attributes of optimized synbiotic mango fruit yogurt-derived bioactive peptides

Bioactive peptides were extracted by ultrafiltration membranes (Vivaspin) of 10 kDa, 5 kDa, and 3 kDa molecular weight cut off using high-speed centrifugation and were tested for antioxidant (by ABTS method) and ACE-inhibitory activity. The degree of proteolysis is highly correlated with these biofunctional activities. In our previous study, yogurt cultures *S. thermophilus* ST 144 and *L. bulgaricus* ssp. *delbrueckii* LB 144 were evaluated for proteolytic activity with 113.47 ± 0.91 μg serine mL^−1^ and 201.11 ± 0.74 μg serine mL^−1^, respectively (Minj et al., 2020). The proteolytic nature of yogurt bacteria could be a useful indicator for the production of small bioactive peptides in the developed synbiotic mango fruit yogurt.

#### 3.4.1 Antioxidant activity of bioactive peptide fractions

The antioxidant activity of the bioactive peptide fraction was evaluated using the ABTS method. Results showed that the antioxidant activity was significantly ( p<0.05 ) higher in the 10 kDa sample than in the 5 kDa and 3 kDa samples. The antioxidant activity was noted as 1,293.09 ± 1.10 mmol/L TEAC in the 10 kDa sample, whereas it was significantly lower, at 1,208.07 ± 2.92 in the 5 kDa sample and 1,047.95 ± 2.20 mmol/L TEAC in the 3 kDa sample (Figure 3C). The 10 kDa bioactive peptide fractions might have higher peptide concentrations, and many peptide sequences could have antioxidant potential. Therefore, the 10 kDa bioactive peptide fractions showed significantly higher antioxidant activity than the other samples. Qian et al. (2011) also reported that the peptidic fractions of 5–3 kDa exhibited good antioxidant activity with the highest in the 10 kDa fraction. A similar study was done on fermented bovine milk peptides and camel milk peptides, which exhibited the highest antioxidant activity in the 5–10 kDa peptide fractions, 110.41–745.35 mM and 844.08–1737.88 mM, respectively (Moslehishad et al., 2013). Bioactive peptide fractions of 5–10 kDa from yogurt whey have also been reported for their high antioxidant activity (Karimi et al., 2021). These research studies agree with our results.

Generally, milk protein sources have reported contradictory results on antioxidant activity. Some researchers reported that the 3 kDa peptide fraction had higher antioxidant activity than the 10 kDa or >10 kDa fractions (Shirkhan et al., 2023; Qian et al., 2011). The low molecular weight peptide fractions have significantly higher ABTS and DPPH free radical scavenging activity (Shirkhan et al., 2023). The milk protein hydrolyzed peptide fraction of <1 kDa demonstrated the highest ABTS radical scavenging activity (Abdel-Hamid et al., 2017; De Gobba et al., 2014). The suggested mechanism of action of low molecular weight peptide could quickly react with fat radicals and prevent lipid peroxidation (Ren et al., 2008).

The ABTS radical scavenging activity and antioxidant activity mainly depend on the peptide sequence, size, molecular mass, amino acid composition, concentration, conformation, and nature of peptides (Karami and Akbari-adergani, 2019; Zou et al., 2016). The antioxidant activity of yogurt peptides can also be affected by the denaturation process and secondary structures (Yuan et al., 2018). The presence of aromatic and hydrophobic amino acid residues in the peptides is a determining factor in the radical scavenging activity. It has also been reported that certain amino acids, such as His (imidazole group), Phe (aromatic amino acid), Pro (hydroxyl radical scavenger), Trp (indolic group), and Tyr (phenolic group), are correlated with the antioxidant activity (Ajibola et al., 2011). These amino acids could be involved in the reduction of oxidative stress by increasing the level of antioxidant enzymes such as glutathione peroxidase (GPx) and superoxide dismutase (SOD). Furthermore, they can also play an important role in minimizing the inflammatory cytokines in adipocytes and eliminating reactive oxygen species.

In another study, antioxidant peptides from goat milk protein fractions exhibited ABTS radical scavenging activity where tyrosine seemed to be a fundamental peptide; however, phenylalanine seemed to play a key role in inhibiting the formation of secondary lipid oxidation products (De Gobba et al., 2014). The presence of hydrophobic amino acids like leucine and valine increases the existence of antioxidant peptides at the interface between the lipid and water phases and increases access to free radicals in the lipid phase (Sarmadi and Ismail, 2010). Overall, it can be stated that different size peptide fractions have different abilities to scavenge free radicals. Therefore, further analysis of the antioxidant peptide profile from the 10 kDa fractions is needed.

#### 3.4.2 ACE-inhibitory activity of bioactive peptide fractions

The bioactive peptide fractions of 3 kDa, 5 kDa, and 10 kDa were evaluated for the ACE-inhibitory activity. Figure 3D illustrates the ACE-inhibitory activity of 10 kDa, 5 kDa, and 3 kDa fractions. The 10 kDa bioactive peptide fraction exhibited significantly higher (82.72% ± 1.24%) ACE-inhibitory activity than the 5 kDa (64.20% ± 1.24%) and 3 kDa (45.68 ± 1.23) fractions. The ACE-inhibitory activity is mainly affected by the presence of specific peptide sequences and probiotic yogurt bacteria, which could be responsible for the production of these bioactive peptide sequences. Korhonen (2009) reviewed the many ACE-inhibitory peptides and their sequences from the milk and milk products. Some of the best-known peptide sequences, Val-Pro-Pro (VPP) and IIe-Pro-Pro (IPP), have been reported for their ACE-inhibitory activity (Nakamura et al., 1995; Sipola et al., 2002). Similar peptide sequences might be present in the 10 kDa peptide fraction and be responsible for the significantly higher ACE-inhibitory activity.

According to Donkor et al. (2007), yogurt made with yogurt culture alone or in combination with probiotic bacteria has significant ACE-inhibitory activity, with higher activity in the probiotic yogurt. The origin of ACE-inhibitory peptides, VPP and IPP, was from *α*_s2_-casein, mainly *κ*- and *β*-casein. The proportion of *α*_s2_-casein varies between ruminants and is considered to comprise up to 10% of casein in bovine milk. The *α*_s2_-casein appears to be readily susceptible to proteolysis and breakdown into small biologically active peptides.

Many bioactive peptides with potential ACE-inhibitory activity have been isolated from yogurt and milk proteins (Gobbetti et al., 2000; Fitzgerald and Murray, 2006). Most of the documented ACE-inhibitory peptides in fermented dairy products are associated with a potential antihypertensive effect. The ACE-inhibitory peptides have a proline residue at the carboxyl-terminal end, are known to be resistant to degradation by digestive enzymes, directly reach the small intestine, and enter the blood circulation in the form of small sequence peptides (Pan et al., 2005). ACE is an important part of the renin-angiotensin system (RAS) and is known for its peripheral blood pressure regulation. This enzyme is responsible for the conversion of angiotensin I into angiotensin II, a potent vasoconstrictor leading to elevated blood pressure. Due to this important role of ACE, the inhibition of this enzyme has been used to treat hypertension (Coppey et al., 2006). Therefore, ACE-inhibitory peptides could play an important role in the reduction of blood pressure through ACE-inhibitory activity.

Fermented milk products, including yogurt, are considered to have a positive effect on high blood pressure. Many studies have shown that fermented milk products made by probiotic bacteria have ACE-inhibitory activity (Chen et al., 2024; Dhakal et al., 2024; Guzmán-Rodríguez et al., 2024; Kim et al., 2021; Ayyash et al., 2018; Ong and Shah, 2008; Donkor et al., 2007). Most of the ACE-inhibitory bioactive peptides are produced by *L. delbrueckii* ssp. *bulgaricus* during milk fermentation from αs1-and β-casein (Yu et al., 2021). Fermentation with *L. delbrueckii* ssp. *bulgaricus* produces ACE-inhibitory peptides (Kim et al., 2021; Sultan et al., 2018; Kliche et al., 2017; Qian et al., 2011). The addition of probiotic culture *L. plantarum* K25 along with yogurt culture increases the ACE-inhibitory activity throughout the 21 days of storage from 22.3% to 49.3% (Min et al., 2020). However, the concentration of ACE-inhibitory peptides could vary depending on the different strains of *L. delbrueckii* used in the yogurt preparation. Based on these studies, probiotic yogurt bacteria NCDC 144 could be considered a potent ACE-inhibitory peptide producer.

Yogurt fortified with prebiotics also affects the production of ACE-inhibitory peptides. Kariyawasam et al. (2021) reported that ACE-inhibitory activity was higher in the synbiotic yogurt than in non-synbiotic yogurt. Similarly, Habibi Najafi et al. (2019) also reported that the presence of prebiotics increased the ACE-inhibitory activity. Yogurt containing prebiotic inulin (Shakerian et al., 2015; Ramchandran and Shah, 2009) and polysaccharides had higher ACE-inhibitory activity (Ramchandran and Shah, 2010). Our finding agrees with published results that found that supplementation with prebiotics increased ACE-inhibitory activity.

The size of the bioactive peptide fraction also has an impact on the ACE-inhibitory activity. According to Schlothauer et al. (2002), the 10 kDa and 50 kDa peptide concentrates had high ACE inhibition, which supports this study. Other studies showed that 3 kDa or <3 kDa fraction had a higher percentage of inhibition (Wu et al., 2019; Rubak et al., 2020); Rubak et al. (2022) reported that ACE inhibition in *Lactococcus lactis* ssp. *lactis* BD17 fermented skimmed goat milk exhibited 57.31%, 24.57%, and 60.33% in <3 kDa, >3 kDa, and supernatant samples, respectively. According to Parmar et al. (2018), goat milk fermented with different lactic acid bacteria has different %ACE inhibition in the peptide fraction. *L. casei* (NK9) fermented goat milk 10 kDa permeates exhibited the presence of AFPEHK ACE-inhibitory peptides. Shukla et al. (2023) reported that fermented camel milk showed higher ACE-inhibitory peptides in 3 kDa permeate fraction. Recently, Devecioglu et al. (2024) reported that the ACE-inhibitory activity of peptide fractions is significantly affected by ultrafiltration. Unfractionated samples had lower ACE-inhibitory activity than fractionated samples. Therefore, ultrafiltration or additional purification increases the ACE-inhibitory activity. The limitation of this study is the identification of ACE-inhibitory peptide sequence in the 10 kDa sample through reverse-phase liquid chromatography-mass spectrometry (RPLC-MS) as well as *in vivo* testing of 10 kDa bioactive peptides to confirm health benefits at the molecular level. This future research direction will provide strength to this study.

## 4 Conclusion

In the present study, the combined effects of three different independent factors (sugar, prebiotic inulin, and mango pulp) were evaluated for their effect on response antioxidant activity. Optimization was successfully conducted by utilizing RSM, and sugar 6%, mango fruit pulp 6.562%, and prebiotic inulin 1% were found to be optimum for the synbiotic mango fruit yogurt preparation. The optimized synbiotic mango fruit yogurt was found to be rich in biofunctional and technofunctional properties, mainly total polyphenol content, total flavonoid content, proteolytic activity, antioxidant, and ACE-inhibitory activity, and best from a microbiological quality point of view. The peptides released from yogurt during the fermentation process exhibited specific bioactivity, with the 10 kDa peptide fraction showing significantly higher antioxidant and ACE-inhibitory activity than the 3 kDa and 5 kDa fractions.

Recently, with increasing interest in health-promoting food, consumers and functional food industries are looking for products that could help prevent lifestyle-related diseases like hypertension, cancer, diabetes, and other metabolic disorders. This research study offers potential for technological and biofunctional advances in synbiotic mango fruit yogurt production. The product could be an excellent candidate to meet the needs of the innovative yogurt development market. However, further research is needed to identify the specific peptide sequences with a metabolomic approach. It is expected that this study will be useful in the application of commercial production of synbiotic mango fruit yogurt with enhanced biofunctional properties. In addition, specific bioactive peptide sequences will be useful in the pharmaceutical industry after a thorough investigation of the bioavailability of these peptides in clinical trials.

## Data Availability

The original contributions presented in the study are included in the article/supplementary material; further inquiries can be directed to the corresponding author.
